# Processing of fish products in Zambia for sustainable food systems and nutritious food for the first 1,000 days of life

**DOI:** 10.3389/fnut.2025.1536097

**Published:** 2025-04-15

**Authors:** Molly B. Ahern, Sven Genschick, Abraham Muluku, Ogan I. Mba, Shakuntala H. Thilsted

**Affiliations:** ^1^Food and Agriculture Organization of the United Nations, Fisheries and Aquaculture Division, Rome, Italy; ^2^Deutsche Gesellschaft Für International Zusammenarbeit (GIZ), Eschborn, Germany; ^3^Ministry of Fisheries and Livestock, Lusaka, Zambia; ^4^Sustainable Agrifood Systems Engineering Laboratory (SASEL), McGill University, Montreal, QC, Canada; ^5^CGIAR, Washington, DC, United States

**Keywords:** fish, nutrient composition, processing, fish powder, fish smoking, sun-dried fish, Zambia, fish value chain

## Abstract

**Background/objectives:**

Fish is a highly perishable food, and in the absence of cold chain technologies, is often preserved using traditional technologies in rural fishing communities of Zambia. High fish losses contribute to food insecurity, malnutrition, and unsustainable food systems in Zambia. The objective of this research was to analyze the nutrient profiles of indigenous fish species and fish products processed using both traditional methods and modern techniques aimed at enhancing food quality, minimizing fish loss and waste, and increasing efficiency. The ultimate goal is to contribute to more environmentally and economically sustainable fish supply chains. We analyzed the proximate, vitamin, mineral and fatty acid composition of ten fish products from inland capture fisheries.

**Methods:**

Samples of ten fish products from inland capture fisheries in Zambia were collected and analyzed for their nutrient content. The potential contribution of each species to recommended nutrient intakes (RNIs) for pregnant and lactating women (PLW) and infants was calculated.

**Results:**

Iron content of fish products ranged from 0.31 to 2.49 mg/100 g, zinc from 0.2 to 1.13 mg/100 g, and calcium from 151.15 to 636.28 mg/100 g. Docosahexaenoic acid (DHA) was high in smoked fish and smoked fish powder processed using improved smoking kilns, which contributed 15–20% of daily DHA requirements for PLW and infants, based on a 25g or 10g serving (respectively). The ten products analyzed contributed 6–33% of daily requirements of DHA for both PLW and infants. Improved techniques for smoking fish resulted in greater content of Vitamins A and E, compared to traditional methods.

**Conclusion:**

This article provides evidence of the nutrient content of various fish products processed using traditional and improved technologies which are important for sustaining fish consumption and contributing to food security and nutrition in Zambia in the first 1,000 days of life.

## 1 Introduction

A significant proportion of undernourished people is found in sub-Saharan Africa, where 22.8% of the population is undernourished (1). Compared to other countries in the region, Zambia has a significantly higher proportion of undernourished people at 46.7%. Additionally, 33.7% of women of reproductive age (WRA) are anemic, and 35% of children under five experience stunted growth, with these issues being more prevalent in rural areas (2). In sub-Saharan Africa, predominantly plant-based diets, with low amounts of animal-source foods contribute to inadequate intakes and poor bioavailability of micronutrients for WRA and young children (3–5).

Fish and other aquatic animals make up 17% of animal protein supply on the African continent (6). Climate variability and shocks lead to seasonal fish scarcity and unsustainable levels of fish loss within the supply chain. Peak production periods often align with the rainy season, causing significant physical and qualitative fish losses (7). This underscores the importance of processing fish during peak production seasons into products that can be consumed during times of scarcity, thus ensuring a sustainable and nutritious food source. Most fish consumed in Zambia comes from inland fisheries, mainly small pelagic species which are usually sun-dried or smoked whole for preservation and marketing. These fish products are highly consumed, especially by the poor (7, 8). However, there is little evidence of the nutritional composition of fish species and processed fish products that are traditionally consumed in Zambia.

Available studies show that the traditional Zambian diet is dominated by plant-source foods and characterized by heavy consumption of staple crops and seasonal scarcity of nutrient-rich foods. The most preferred meal is “nshima” (a thick maize porridge) consumed with small portions of seasonal side dishes (9, 10). In these side dishes, fish is a common animal-source food, making up 26.4% of animal protein in the Zambian diet (11) and consumed by 41% of households (in comparison to 28% consuming meat, 25% eggs and 25% milk or other dairy products) in the preceding 24 hours of the 2009 National Nutrition Surveillance household survey (12). Just 13% of children under 2 years of age in Zambia consume adequate diets (2) due to monotonous, staple-heavy diets and lack of caretakers’ nutrition knowledge (3, 13, 14).

Fish possess significant potential to enhance nutrition in the first 1,000 days of life, through food-based interventions due to their richness in essential fatty acids, vitamins, and minerals such as iron, zinc, calcium, vitamin A, and vitamin B12, which are often identified as “problem nutrients” (68). This period spans from conception to the child’s second birthday, and is recognized as a crucial time for physical growth and cognitive development in children. This period is especially important for maternal and child nutrition. This period also covers the recommended transition from exclusive breastfeeding in the first 6 months of life to the introduction of complementary foods, along with continued breastfeeding. The World Health Organization (WHO)/United Nations Children’s Fund (UNICEF) Global Strategy for Infant and Young Child Feeding makes the following recommendation:

“…low-cost complementary foods, prepared with locally available ingredients using suitable small-scale production technologies in community settings, can help to meet the nutritional needs of older infants and young children (15).”

Commercially processed plant-based fortified complementary foods are available in Zambia’s formal markets, however, they are not available or affordable to rural communities where the prevalence of malnutrition is highest (16). Home-based approaches to complementary feeding are promoted by the National Food and Nutrition Commission of Zambia (NFNC) as well as the Ministries of Health, Agriculture and Fisheries as sustainable food-based solutions to combat malnutrition (17–21). Despite the recognition of the nutritional importance of fish for cognitive development in the first 1,000 days of life (22); the increased fatty acid content of breastmilk due to fish consumption by mothers (23) and the evidence that children consuming fish are less likely to be stunted (5), the promotion of fish and fish products during this critical period of growth is still not common. The lack of inclusion of fish in complementary foods is often due to concerns over bones as a choking hazard (24, 25). Fish processing innovations such as powdering fish offer an opportunity to extend the consumer base to non-traditional consumers such as infants and young children. In addition, powdering of fish contributes to reduction of loss and waste by utilizing the whole fish, and increased vitamin and mineral content through inclusion of parts such as bones which are a source of calcium. In addition, there is evidence of acceptability and effectiveness of complementary foods containing fish, however, these studies were primarily focused on Asian populations (24, 26, 27), with one in Zambia and Malawi (28).

The study aims to fill gaps in data by providing evidence of the nutrient composition of small indigenous species from rural Zambia in their fresh, dried, smoked and powdered forms which are important for food security and nutrition in rural Zambia. This study also aims to fill a gap in knowledge on impact of processing methods on nutrient retention by offering some analysis of retention of nutrients across traditional versus improved processing methods. This study presents the results of nutrient analyses of two local, nutrient-rich small fish species from Northern Province, Zambia. These species were processed into various fish products using both traditional and improved processing methods, specifically targeting the first 1,000 days of life. The fish products were produced through various small-scale processing techniques commonly found in local areas, including traditional sun-drying, traditional fish smoking (*ubusani* method), and the powdering of dried or smoked fish using a mortar and pestle. Fish products were also made using “improved” technologies like fish smoking kilns, small solar drying tents, and hammer mills. These technologies aim to improve the quality and safety of fish products, increase efficiency, reduce fish loss and waste, and contribute to environmentally and economically sustainable fish supply chains. These “improved” technologies have been acknowledged for their social, economic, and environmental sustainability. The resulting fish products are acceptable to local communities, and the equipment is relatively inexpensive to construct and maintain. Additionally, these technologies use less fuel (such as firewood for smoking fish) and reduce fish losses (69).

## 2 Materials and methods

### 2.1 Sampling

**Figure 1:**
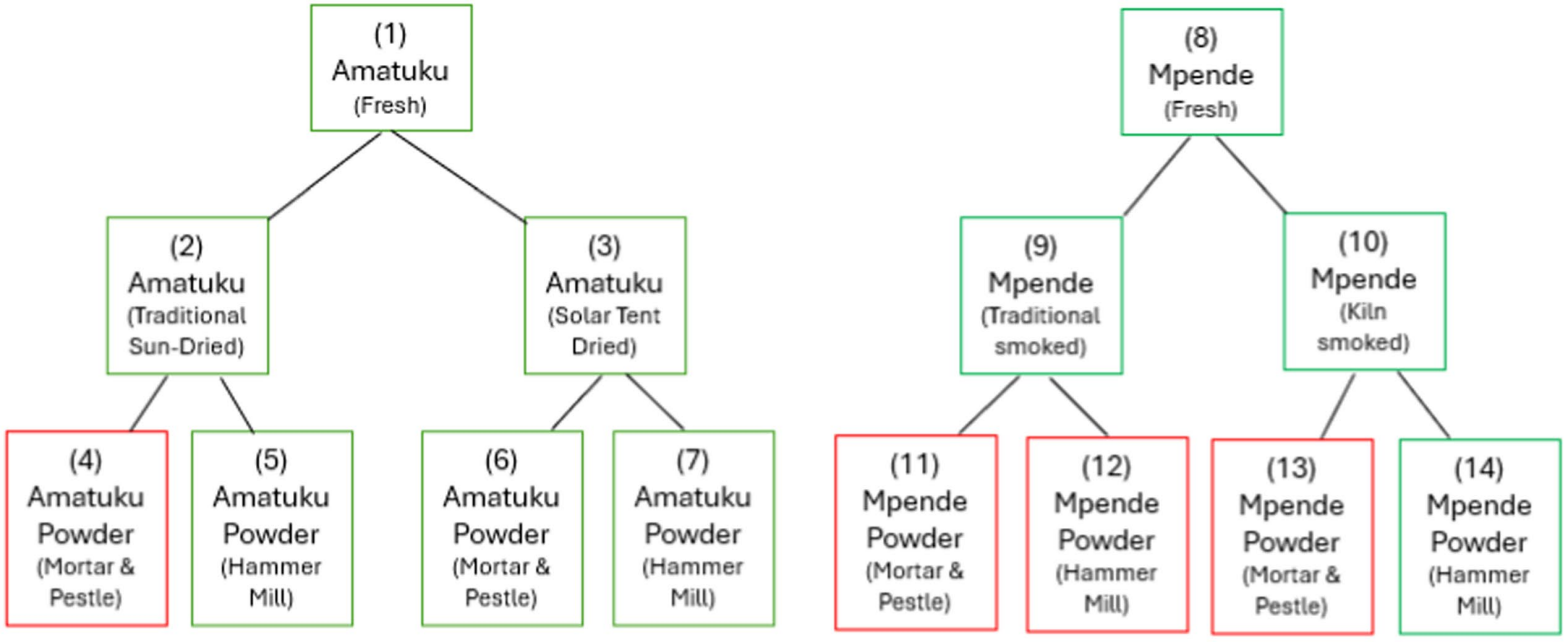
Sampling protocol for two fish species, processed to fish powder, using local and test methods. Those outlined in green were analyzed for nutrients, while those in red were not fully processed (due to issues experienced with sample collection and time spent processing using mortar and pestle) nor sent for nutrient analysis.

Sampling of fish at different stages of the value chain—from fresh fish to dried or smoked fish and fish powder products—were done at the project site(s). Fish species were selected based on availability of species within proximity and locally accepted processing methods for each species. The local fisherfolk informed the research team that *Amatuku, Imintesa*, and *Imishipa* are usually preserved by sun-drying, whereas *Imilonge* and *Mpende* are typically preserved by smoking. Due to availability, the fish species selected for sun-drying/solar tent drying was *Amatuku* (*Tilapia sparmanii*) and for smoking and kiln smoking, *Mpende* (*Tilapia rendalli*). The fish were cleaned according to local practices prior to sampling and further processing of fresh samples. The design of the sampling protocol used in this study is shown in Figure 1, and a description of each sample is included in Table 1.

**Table 1:** Sample descriptions.

Species name	Local name	Form	Primary processing method	Secondary processing method	Summary name
*Tilapia sparrmanii*	*Amatuku*	Fresh	None	None	Fresh *Amatuku*
		Dried whole fish	Open Sun-dried	None	Trad. Dry *Amatuku*
		Dried whole fish	Solar Tent dried	None	Tent Dry *Amatuku*
		Powdered fish	Open Sun-dried	Powdered using hammer mill	*Amatuku* Powder (Trad. Dry / Milled)
		Powdered fish	Open Sun-dried	Powdered using mortar & Pestle	*Amatuku* Powder (Trad. Dry / M&P)
		Powdered fish	Solar Tent Dried	Powdered using hammer mill	*Amatuku* Powder (Tent Dry / Milled)
*Tilapia rendalli*	*Mpende*	Fresh	None	None	Fresh *Mpende*
		Smoked whole fish	Traditional *Ubusani* smoked	None	Trad. Smoked *Mpende*
		Smoked whole fish	Kiln smoked	None	Kiln Smoked *Mpende*
		Powdered fish	Kiln smoked	Powdered using hammer mill	*Mpende* Powder (Kiln Smoked / Milled)

**Figure 2:**
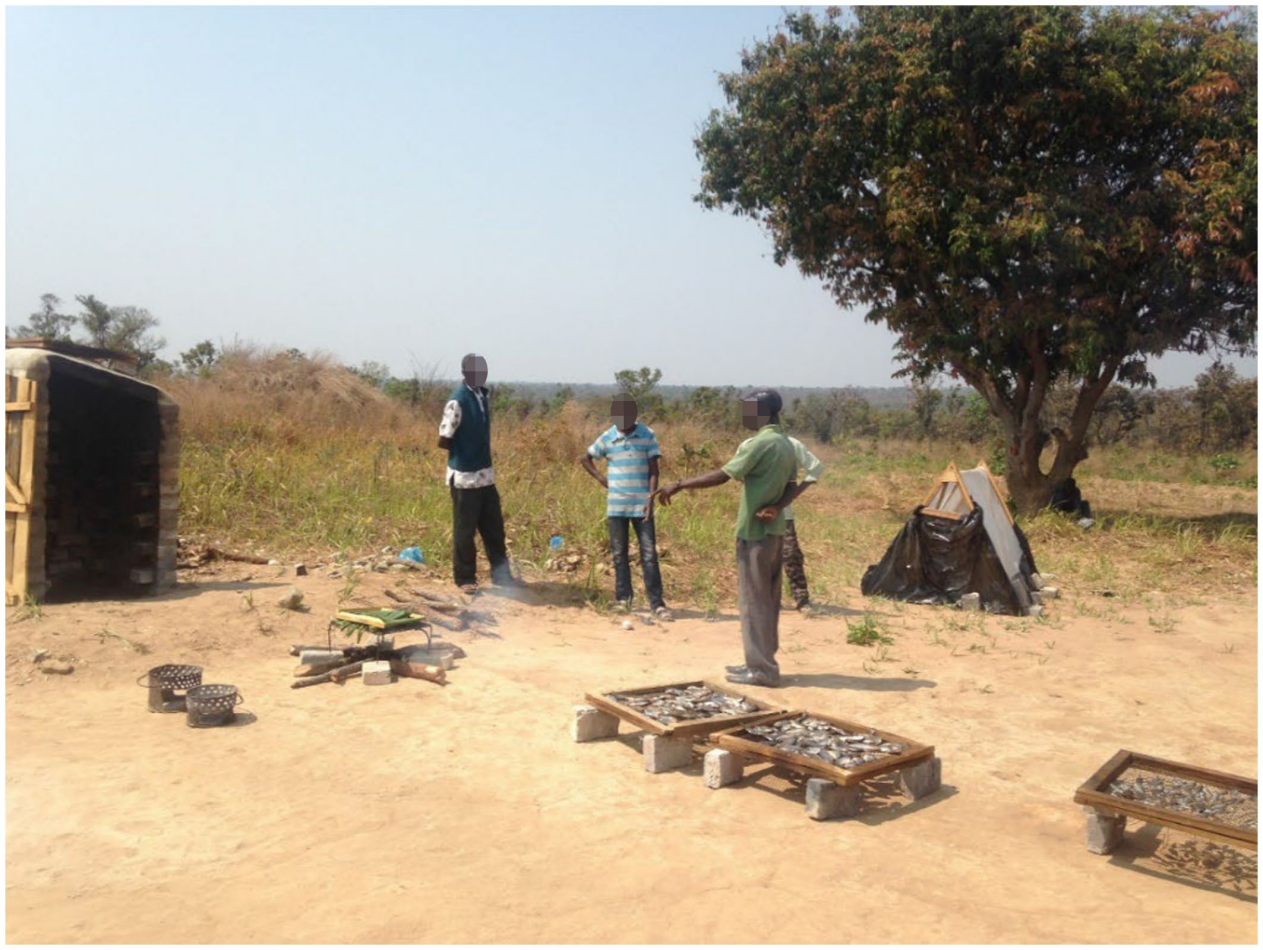
Sample processing site in Ipusukilo Ward, Northern Province Zambia, where two fish smoking techniques (kiln and *ubusani*) and two fish drying techniques (solar tent and traditional sun-drying) were conducted.

Samples were obtained on the 26th of September 2017 from local fishermen in Nsombo, a village on the northern side of Lake Banguwelu, within Luwingu District, Northern Province. Both fish species were bought for USD 3.50 per kilogram, washed, sorted, and placed on ice in cooler boxes by local fishermen before being transported to the processing site in Ipusukilo Ward, Luwingu District, Northern Province (see Figure 2). At the processing site, local fish processors cleaned the samples to obtain raw, edible parts by removing gills and intestines. Ceramic knives, plastic cutting boards, and plastic buckets were used to avoid contamination. Then, the raw, cleaned fish were packed or processed further into dried and smoked fish as well as fish powder.

Fresh Sample Preparation (Raw Edible Parts)—At the collection site, raw, cleaned fish samples were placed into plastic bags and then stored in a freezer at −18°C. These samples were kept at this temperature in the field office of Self Help Africa, located in Luwingu, Zambia. Subsequently, they were packed in an insulated box lined with ice packs and transported to Lusaka. From there, they were sent by air transport to McGill University in Montreal, Canada.

Primary Processing (Drying and Smoking)—Fish samples were subjected to sun-drying using two methods: traditional open sun-drying and solar tent drying. A small solar tent was set up to prevent contamination of samples with dust and pests. Both the open sun-drying and solar tent drying processes took 2 days, achieving a desirable level of dryness as verified by local community members. For smoked fish samples, the fish were smoked using either the traditional method (*ubusani*) or a newly installed smoking kiln. Upon confirmation from local community members that the samples were adequately smoked, they were packed in plastic bags.

Secondary Processing (Powdering)—Fish samples were randomly selected from sun-dried or solar tent-dried fish. They were powdered using two methods: (1) a wooden mortar and pestle, and (2) a hammer mill. The fish powder was processed by pounding with a mortar and pestle. It was then sieved using a fine sieve similar to those used in households for making maize meal. Larger pieces were pounded again and sieved. This process was repeated until all fish samples were converted into powder, minimizing waste. Only one sample powdered using the mortar and pestle was included for analysis, due to issues with sample collection and processing (there was insufficient quantities of fish for all samples, and due to the time needed for fish processors to grind fish into powder using mortar and pestle, other samples were prioritized). An improved method for processing fish powders was tested using a hammer mill suitable for small-scale production. The hammer mill used was provided by an agricultural machinery supplier in Lusaka. The fish was processed through the hammer mill using a fine sieve setting to produce fish powder.

### 2.2 Analysis

Analytical Methods: The frozen samples were air-shipped from Lusaka, Zambia to Montreal, Canada, where McGill University conducted the nutrient analysis. The samples were kept on ice during transport and then moved to a −80°C laboratory freezer upon arrival at the Food and Bioprocess Engineering laboratory, Macdonald Campus, McGill University, Canada, until analyses were conducted. Standard analytical methods were employed for assessing each class of nutrients in the fish samples. The analyses conducted, along with the referenced methodologies, are summarized in Table 2 below. All the analyses were conducted in triplicates and the nutrients composition presented as mean and standard deviation. One-way ANOVA was also performed to compare nutrient differences among the fish samples.

**Table 2:** Analyzed nutrients.

Class	Nutrient	Unit - /100 g of product (fresh fish – raw edible parts; smoked fish; dried fish; or fish powder)		Model of instrument	Calibration method	Method reference
Proximates	Protein	g/100 g		Dumas Nitrogen Analyzer, NDA 701 (Velp Scientifica, Italy)	Calibration curve with 6 replicates of EDTA	Dumas Combustion Method (AOAC 992.15)
	Fat	g/100 g		SER 148 Solvent Extraction (Velp Scientifica, Italy)	Blank extraction using petroleum ether	Randall technique (AOAC 963.15)
	Moisture	g/100 g		Isotemp Air Oven (Fisherbrand)	NIST thermometer inside the oven set at 105°C	Air drying (AOAC 950.46)
	Ash	g/100 g		Eurotherm 3,216 furnace (Thermolyne)	High precision thermocouple with the furnace set at 550°C	Direct Method (AOAC 920.153)
Minerals	Total Fe	mg/kg		ICP-MS Spectrometer Varian 820 MS (Analytik Jena AG, Jena, Germany)	Certified Reference Material (CRM): NIST 1547 (Peach leaves)	ICP-Mass Spectrometry Method (ICP-MS) (75)
	Haem Fe	mg/kg				
	Non-Haem Fe	mg/kg				
	Zinc	mg/kg				Acid Digest, ICP OES (63)
	Calcium	mg/kg				Acid Digest, ICP OES (63)
	Iodine	μg/kg				TMAH Digestion, ICP MS (63)
	Selenium	mg/kg				TMAH Digestion, ICP MS (63)
	Phosphorus	mg/kg				Acid Digest, ICP OES (63)
	Magnesium	mg/kg				Acid Digest, ICP OES (63)
	Sodium	mg/kg				Acid Digest, ICP OES (63)
	Potassium	mg/kg				Acid Digest, ICP OES (63)
	Manganese	mg/kg				Acid Digest, ICP OES (63)
	Sulphur	mg/100 g				Acid Digest, ICP OES (63)
	Copper	mg/kg				Acid Digest, ICP OES (63)
	Chromium	mg/kg				Wet Oxidation, ICP MS (63)
Vitamins	Vitamin B	B12	μg/100 g			Surface plasmon resonance (AOAC 2011.16)
	Vitamin D	D3	IU/100 g	1,260 Infinity II HPLC (Agilent Technologies) coupled with DAD	Baseline & blank check followed by preparation of calibration curves using 5 different concentrations of corresponding standards to analyte.	HPLC (64)
		D2	IU/100 g			HPLC (64)
		250HD3	μg/kg			HPLC (65)
	Vitamin E	E (α-tocopherol)	IU/100 g			HPLC (64)
		E (γ, δ tocopherols)	IU/100 g			HPLC (64)
	Folate	Folate	μg/kg			Optimal biosensor assay (AOAC 2011.05)
	Vitamin A All-trans retinol (μg) 13-cis retinol (μg)	Vitamin A2 -all-trans-3,4-dehydroretinol	μg/kg			HPLC (66)
	All-trans 3,4-dehydroretinol (μg)	b- carotene				
		13-cis-retinol				
	13-cis 3,4-dehydroretinol (μg)	13-cis-dehydroretinol				
	β-carotene (μg)**	All-trans retinol				
	Total vitamin A (RAF) ††	All-trans dehydroretinol				
Fatty acids			mg/100 g	GLC 7650 (G4567A) coupled with FID (Agilent Technologies)	Calibration curve using certified reference material-Supelco 37 FAME mix; running blank injection and injecting standard solutions	GLC (67)

All proximate components and minerals were analyzed in triplicates and the results are presented in Table 3. Energy was calculated using Atwater factors from assayed proximate components (29). All results are presented based on 100 g of the product tested. A selection of vitamins, minerals and fatty acids are presented in Tables 4–6, and the full results from laboratory analysis of each sample are available in the Supplementary materials.

**Table 3:** Proximate composition of different processed forms of two fish species.

Fish species	Sample	Crude Fat	Crude Protein	Ash	Moisture	Energy
		g/100 g	g/100 g	g/100 g	g/100 g	kJ/100 g
*Amatuku*	Fresh	3.51 ± 0.14	16.17 ± 0.20	4.04 ± 0.00	75.74 ± 1.70	414
	Trad. Dry	11.26 ± 0.22	56.96 ± 0.61	14.16 ± 0.28	10.53 ± 0.06	1,505
	Tent Dry	12.13 ± 0.39	56.14 ± 0.39	13.13 ± 0.42	10.70 ± 0.11	1,537
	Powder (Trad. Dry / Milled)	9.90 ± 0.09	53.19 ± 0.12	17.62 ± 0.38	7.72 ± 0.05	1,467
	Powder (Trad. Dry / M&P)	10.88 ± 0.07	57.93 ± 0.83	14.92 ± 0.48	11.20 ± 0.10	1,474
	Powder (Tent Dry / Milled)	10.30 ± 0.07	53.41 ± 0.59	15.48 ± 0.17	10.44 ± 0.05	1,465
*Mpende*	Fresh	4.35 ± 0.21	14.01 ± 0.18	3.39 ± 0.09	79.19 ± 0.53	383
	Trad. Smoked	14.28 ± 0.34	59.68 ± 0.67	16.89 ± 0.42	1.61 ± 0.09	1,671
	Kiln Smoked	13.19 ± 0.38	58.83 ± 0.38	17.49 ± 0.56	8.17 ± 0.02	1,528
	Powder (Kiln Smoked / Milled)	12.03 ± 0.23	59.37 ± 0.07	17.49 ± 0.25	1.62 ± 0.03	1,616

Results for fresh fish samples are presented as g / 100 g of raw, edible parts, while results for all other samples are expressed on wet weight basis.

**Table 4:** Mineral composition of different processed forms of two fish species.

Fish species	Sample	Ca mg/100 g	Zn mg/100 g	Fe mg/100 g	Se μg/100 g
*Amatuku*	Fresh	182.73 ± 3.08	0.34 ± 0.03	0.31 ± 0.01	1.74 ± 0.14
	Trad. Dry	530.27 ± 1.97	0.98 ± 0.05	2.27 ± 0.00	6.38 ± 0.81
	Tent Dry	546.66 ± 2.27	1.02 ± 0.02	2.48 ± 0.13	6.10 ± 0.36
	Powder (Trad. Dry / Milled)	550.45 ± 1.12	1.13 ± 0.06	1.56 ± 0.03	6.79 ± 0.86
	Powder (Trad. Dry / M&P)	453.65 ± 2.75	1.13 ± 0.03	2.49 ± 0.04	6.82 ± 0.73
	Powder (Tent Dry / Milled)	483.17 ± 1.52	1.02 ± 0.04	1.58 ± 0.00	7.22 ± 0.61
*Mpende*	Fresh	151.15 ± 0.54	0.20 ± 0.01	0.40 ± 0.01	1.55 ± 0.07
	Trad. Smoked	487.25 ± 3.00	0.90 ± 0.01	2.29 ± 0.00	6.81 ± 0.38
	Kiln Smoked	583.06 ± 4.17	0.90 ± 0.05	1.43 ± 0.01	6.53 ± 0.73
	Powder (Kiln Smoked / Milled)	636.28 ± 1.25	0.93 ± 0.03	2.40 ± 0.00	6.48 ± 0.54

**Table 5:** Vitamin composition of different processed forms of two fish species.

Fish species	Sample	Retinol (A)	Cyanocobalamin (B12)	Total Vit. D	Total Vit. E
		μg/100 g	μg/100 g	μg/100 g	μg/100 g
*Amatuku*	Fresh	0.99 ± 0.00	0.03 ± 0.00	0.08 ± 0.00	3.47 ± 0.06
	Trad. Dry	1.50 ± 0.20	0.09 ± 0.00	0.01 ± 0.00	0.78 ± 0.00
	Tent Dry	nd	nd	0.16 ± 0.00	5.44 ± 0.05
	Powder (Trad. Dry / Milled)	0.83 ± 0.00	0.09 ± 0.00	0.19 ± 0.03	2.41 ± 0.01
	Powder (Trad. Dry / M&P)	0.85 ± 0.01	0.10 ± 0.00	0.04 ± 0.00	1.41 ± 0.10
	Powder (Tent Dry / Milled)	1.07 ± 0.06	0.09 ± 0.00	0.03 ± 0.00	1.81 ± 0.00
*Mpende*	Fresh	0.71 ± 0.00	0.02 ± 0.00	0.05 ± 0.00	1.86 ± 0.01
	Trad. Smoked	0.89 ± 0.01	0.09 ± 0.00	0.22 ± 0.00	2.46 ± 0.01
	Kiln Smoked	1.07 ± 0.01	nd	0.22 ± 0.01	3.18 ± 0.00
	Powder (Kiln Smoked / Milled)	1.04 ± 0.00	nd	0.15 ± 0.00	3.05 ± 0.01

**Table 6:** Fatty acid composition of different processed forms of two fish species.

Fish species	Sample	∑SFA mg/100 g	∑MUFA mg/100 g	C18:2n-6c [LA] mg/100 g	C18:3n-3 [ALA] mg/100 g	C20:4n-6 (AA) mg/100 g	C20:5n-3 [EPA] mg/100 g	C22:6n-3 [DHA] mg/100 g	∑PUFA mg/100 g
*Amatuku*	Fresh	55.94 ± 1.08	483.06 ± 7.34	33.77 ± 0.28	10.80 ± 0.30	7.84 ± 0.51	14.86 ± 0.15	44.43 ± 5.43	147.57 ± 8.52
	Trad. Dry	137.98 ± 1.80	612.66 ± 12.99	44.00 ± 0.71	19.23 ± 0.14	15.94 ± 0.53	36.91 ± 0.94	66.88 ± 5.34	244.21 ± 10.08
	Tent Dry	180.93 ± 7.96	979.60 ± 5.38	61.67 ± 1.86	39.54 ± 0.80	15.42 ± 1.31	8.80 ± 0.65	78.28 ± 13.35	260.99 ± 22.84
	Powder (Trad. Dry / Milled)	140.98 ± 6.08	566.88 ± 26.00	39.81 ± 1.82	21.19 ± 0.79	13.43 ± 0.86	31.32 ± 1.60	63.69 ± 10.53	217.91 ± 18.64
	Powder (Trad. Dry / M&P)	100.69 ± 4.75	668.94 ± 24.87	43.47 ± 3.66	25.44 ± 1.15	16.66 ± 0.83	36.35 ± 1.46	66.40 ± 5.66	250.10 ± 16.89
	Powder (Tent Dry / Milled)	149.57 ± 4.23	616.57 ± 10.58	46.28 ± 1.61	25.62 ± 0.65	17.01 ± 0.82	5.55 ± 0.67	66.58 ± 4.93	220.60 ± 10.11
*Mpende*	Fresh	54.75 ± 4.94	501.83 ± 7.81	20.30 ± 1.77	8.20 ± 0.15	4.93 ± 0.48	11.08 ± 0.45	52.40 ± 0.92	115.45 ± 4.56
	Trad. Smoked	147.24 ± 4.67	1032.85 ± 10.70	74.51 ± 0.98	12.72 ± 0.83	12.04 ± 2.25	45.52 ± 3.27	157.29 ± 12.16	333.18 ± 22.49
	Kiln Smoked	104.93 ± 1.12	723.05 ± 4.51	54.17 ± 0.18	24.00 ± 0.32	18.18 ± 0.53	39.85 ± 0.71	167.63 ± 1.95	350.26 ± 12.43
	Powder (Kiln Smoked / Milled)	138.92 ± 2.67	970.95 ± 20.02	92.78 ± 4.85	33.64 ± 0.82	5.62 ± 0.38	8.61 ± 0.58	173.22 ± 15.99	346.95 ± 24.09

SFA, saturated fatty acid; MUFA, monounsaturated fatty acid; PUFA, polyunsaturated fatty acid; LA, linoleic acid; ALA, α-linolenic acid; EPA, eicosapentaenoic acid; DHA, docosahexaenoic acid. nd, not detected. Limit of detection for all fatty acids: 6 mg/100 g.

**Figure 3:**
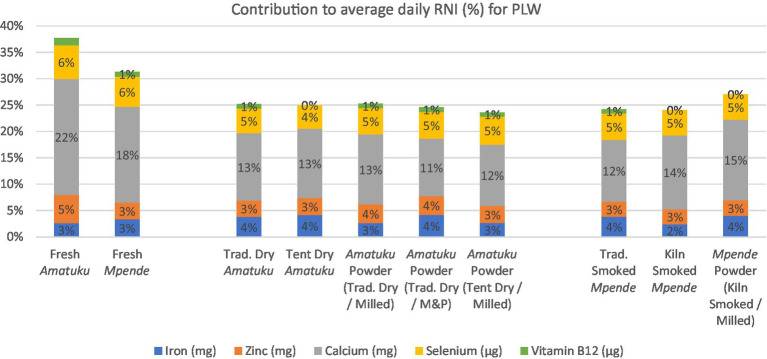
Average daily RNI of 5 key vitamins and minerals for PLW and contribution (%) to RNI for each fish product analyzed based on standard serving of fish products for PLW, as determined by the National Food and Nutrition Commission (NFNC) (70), is 125 g of fresh fish, which is approximately 25 g of dried / smoked fish products.

**Figure 4:**
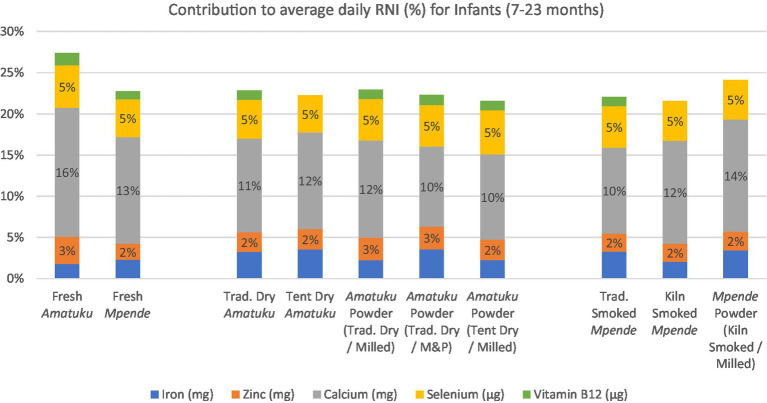
Average daily RNI of 5 key vitamins and minerals for infants age 7–23 months and contribution (%) to RNI for each fish product analyzed based on standard serving of fish products for infants, as determined by the National Food and Nutrition Commission (NFNC) (70) is 40 g of fresh fish, which is approximately 8-10 g of dried/smoked fish products.

Vitamins and minerals were analyzed in triplicates and presented in Tables 4, 5 as per analytical results. Species analyzed for vitamins D and E were reported in the International System of Units (SI) units (μg/100 g). For some samples, a result of ‘none detected’ is given when a quantifiable result was found for one replicate, but the corresponding replicate(s) returned a result below the LOQ. The composition of nutrients of public health significance, vitamins and fatty acids, in relation to RNI’s are shown in Figures 3, 4 and discussed in the discussion section. A total of 27 fatty acids as well as total MUFAs (monounsaturated fatty acids), SFAs (saturated fatty acids) and PUFAs (polyunsaturated fatty acids) were analyzed. Select fatty acid components are presented in Table 6 as per analytical results.

The potential contribution of each species to RNIs of nutrients of interest during the first 1,000 days of life was calculated first by assigning an average RNI target for each nutrient based on RNIs detailed in (30). These guidelines were selected over more recently published RNI guidelines as they establish global nutrient requirements, based on research and information from around the world, rather than more recently published dietary reference values that are more regionally (31) or nationally specific (32, 33). An average RNI target was calculated for PLW, to account for variations in requirements throughout the three trimesters of pregnancy and first 12 months of lactation, and for infants to account for variations in requirements throughout the period from 7 to 23 months (30). Then, we calculated the contribution from a standard portion of each sample as a percentage of the average RNI. The standard portion was determined based on the National Food and Nutrition Commission (NFNC) (70), which showed that women aged 15–49 years who were considered ‘moderate fish consumers’ (50th percentile) consumed 125 g of fresh fish (for freshwater fish), which is approximately 25 g of dried / smoked fish products. The same survey showed that infants aged 0–35 months who were considered ‘moderate fish consumers’ (50th percentile) consumed 41 g of fresh fish, which is 8-10 g of dried / smoked fish products.^1^ The key nutrients discussed here include iron, zinc, calcium, iodine, vitamin A, and vitamin B12. The RNI for iron and zinc vary further according to the estimated overall dietary bioavailability. This bioavailability is influenced by several factors, including the presence of flesh foods, phytates, and other elements. Consequently, RNIs are provided based on different dietary bioavailability categories. The typical Zambian diet in the targeted region consists of milled maize flour, fish, and vegetables. This diet is classified under the ‘10% bioavailability’ category for iron and the ‘moderate bioavailability’ category for zinc (30).

An extensive search was conducted for existing data on the nutrient composition of *Tilapia rendalli* and *Tilapia sparmanii,* including their sun-dried, smoked, or powdered forms. The search yielded limited results, prompting an expansion to include similar species from the region as well as a review of global food composition databases on fish and shellfish. The Zambia National Food and Nutrition Commission (NFNC) food composition tables book has all tilapia species aggregated for food composition data, lacking details on the multitude of tilapia species commonly consumed in the country (19). Nölle et al. (34) conducted an analysis of samples from both *Tilapia rendalli* and *Tilapia sparmanii*. They examined the fish in their fresh forms, as well as in smoked (*Tilapia rendalli*) and sun-dried (*Tilapia sparmanii*) forms. However, they did not include an analysis of fish powders. Longwe and Kapute, (35) analyzed pond-raised *Tilapia rendalli* in Malawi, both in smoked and sun-dried forms. Other studies, including Chetty et al. (71), have analyzed specific nutrients such as the fatty acid content of *Tilapia sparmanii.* However, their study focused solely on fish muscle tissues, excluding heads, bones, and viscera. Consequently, a direct comparison between the whole fish samples analyzed in our study and the fillet-only samples from Chetty et al.’s research is not provided here. The FAO/INFOODs uFish 1.0 database on fish and shellfish is a global database on the nutrient composition of fish and shellfish. It currently includes analyses of fresh fish species, but not dried, smoked, powdered, or other forms. Most of the species represented are those that are commercially traded internationally and often only include fillets. One tilapia species included in the database is *Oreochromis niloticus*. Analyses of fish powders produced in Zambia (36) as well as fish powders from Malawi, specifically from usipa or *Engraulicypris sardella*, were utilized for the development of the Malawi Food Composition Table (37). These analyses were used for comparative purposes.

## 3 Results

### 3.1 Proximate composition

The energy, protein, fat, moisture and ash composition of all 10 fish products are shown in Table 3. The total energy content varied, with a range of 383–1,615 kJ/100 g which is related to variation in fat content in the two species, and the density by weight of the different products. Nutrient density per 100 g of fresh fish products is lower than nutrient density per 100 g of dried, smoked, and powdered fish products due to reduction of moisture content in these products.^2^ The total protein content in fish species ranged from 14.01 to 59.68 g/100 g. The fat content ranged from 3.51 to 14.28 g/100 g. Ash content ranged from 3.39 to 17.62 g/100 g. The moisture content of fish species ranged from 1.61 to 79.19 g/100 g (see Table 3). While the moisture content of dried *Amatuku* products (traditional and solar tent dried) is similar, the fish powders processed using the hammer mill have a notably reduced moisture content in comparison to those processed using mortar and pestle.

### 3.2 Mineral composition

As shown in Table 4, iron content varied with a range from 0.31 to 2.49 mg/100 g. However, the iron content is significantly higher per 100 grams in dried, smoked, and powdered fish products, as expected as dried products are more nutrient dense by weight, due to reduction of moisture content. Zinc concentration varied from 0.2 to 1.13 mg/100 g. Calcium content ranged from 151.15 to 636.28 mg/100 g. The selenium content in fresh fish analyzed here ranged from 1.55–1.74 μg/100 g. Selenium content of dried, smoked and powdered fish samples ranged from 6.10–7.22 μg/100 g.

### 3.3 Vitamin composition

Table 5 presents the vitamin composition of the ten fish samples analyzed. Vitamin A (Retinol) content of fish products analyzed in this study ranged between 0.83 and 1.50 μg/100 g, and was undetected in 1 sample (solar tent dried *Amatuku*). The fish products in this study had vitamin B12 levels ranging from 0.02 to 0.10 μg/100 g, with three samples showing no detectable levels. Total Vitamin D concentrations varied between 0.05 to 0.22 μg/100 g among the ten fish products analyzed. Vitamin E in the form of *α*-tocopherol, *δ*-tocopherol and *γ*-tocopherol was analyzed in all ten fish products, as well as total vitamin E. Across all 10 fish products analyzed, α-tocopherol was undetected in 9 of the products, with only the fresh *Amatuku* having a detectable result (0.42 μg/100 g), and γ-tocopherol was not detected for any of the fish samples (see Supplementary materials). On the other hand, δ-tocopherol was detected for all 10 fish products analyzed, ranging from 0.78 to 5.44 μg/100 g (see Supplementary materials).

Based on a 125 g serving of fresh fish, or a 25 g serving of dried fish, all fish samples would provide less than 5% of RNI for iron, zinc, and selenium for PLW and infants (Figures 3, 4). All the dried and smoked fish products would contribute 10–15% of RNI for calcium for PLW and infants, while fresh fish samples contribute 13–22%. The contribution of the fish products tested in this study to RNI for PLW and infants for Vitamin B12 was less than 2%, and for Vitamin A, it was nearly 0%.

### 3.4 Fatty acid composition

A summary of total SFAs, MUFAs, PUFAs and select fatty acids (LA, linoleic acid; ALA, *α*-linolenic acid; AA, arachidonic acid; EPA, eicosapentaenoic acid; DHA, docosahexaenoic acid) is included in Table 6. Total PUFA, monounsaturated fatty acid (MUFA) and saturated fatty acid (SFA) contents ranged from 54.75 to 1,032.85 mg/100 g.

**Figure 5:**
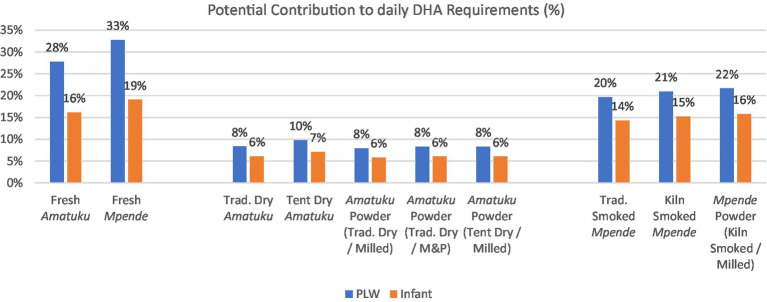
Potential contribution to daily nutrient requirement^a^ of DHA from a standard serving^b^ of each fish product for PLW and infants (7–23 months). (M&P = mortar & pestle used to powder the fish). ^a^ Daily average nutrient requirement of DHA for PLW is 200 mg/day and the adequate intake for infants is 10–12 mg/kg/day (62). For infants, an average figure of 110 mg/day is used. This was calculated by taking the midpoint within the maximum range of adequate intakes throughout the age period (10 mg/kg/day for a 7-month-old of 7.6 kg and 12 mg/kg/day for a 23-month-old of 12.0 kg) where weight is estimated at the 50th percentile according to WHO growth standards (76). ^b^ Standard serving of fish products for PLW, as determined by the National Food and Nutrition Commission (NFNC) (70), is 125 g of fresh fish, which is approximately 25 g of dried / smoked fish products. Standard serving of fish products for infants, as determined by the National Food and Nutrition Commission (NFNC) (70) is 40 g of fresh fish, which is 8-10 g of dried/smoked fish products.

The percentage contribution to daily average nutrient requirement of DHA for PLW and infants (7–23 months) from a standard portion of fish is shown in Figure 5, demonstrating that all fish products tested, would contribute 6–33% of daily requirements of DHA for both PLW and infants. Interestingly, all three of the samples of smoked *Tilapia rendalli* (including fish powder made from smoked *Tilapia rendalli*) provide 15–20% of daily requirements of DHA for both PLW and infants based on a 25 g (PLW) or 10 g serving (infants).

### 3.5 Analysis of improved processing methods

The number of samples of each product were insufficient to make any statistical comparisons of the effect of processing methods on the nutrient composition of fish. However, it is possible to observe that the vitamin A, vitamin E and protein content of solar-tent dried *Amatuku* is greater than the open-sun dried (traditional) method of drying fish. Likewise, the vitamin A, vitamin E, Calcium, Iron and DHA content of kiln-smoked *Mpende* is greater than the traditional-smoked *Mpende*. Further studies can investigate the impact of improved technologies on the nutrient composition of dried and smoked fish products by collecting and analyzing further samples from various locations and fish processors using the same technologies.

### 3.6 Limitations of this study

A small size of pooled samples was used in this study. Challenges encountered following the design of the sampling protocol led to the non-collection of certain samples for nutrient analysis. Consequently, this has resulted in an incomplete assessment of nutrient retention changes across different processing methods. Given the local context and resource limitations, it was not feasible to obtain larger and more representative sample sizes. While it is recognized that these are limitations of the study, given the lack of existing data on nutrient composition of fish species in Zambia, the results are still of significant value, providing location specific estimates for comparison with future analyses.

## 4 Discussion

### 4.1 Proximate composition

The total protein content in fish species ranged from 14.01 to 59.68 g/100 g (Table 3) and can be assumed to be of high dietary quality, being an animal-source protein (38). Fat content ranged from 3.51 to 14.28 g/100 g, and is known to vary much more widely than other proximate components of fresh fish, and usually reflects differences in the way fat is stored in particular species but may also be affected by seasonal/lifecycle variations and the diet/food availability of the species at the time of sampling (39). The large variation in ash content (3.39 to 17.62 g/100 g) is related to inclusion of bones as edible parts, or due to various processing methods used in primary and secondary processing, which would lead to higher ash content. The moisture content of fish species ranged from 1.61 to 79.19 g/100 g (see Table 3). Moisture content and water activity in dried fish products are critical because it gives information on the safety and stability of the product with respect to microbial growth, chemical and biochemical reactions, and physical properties (40). Moisture content below 10 g/100 g is the standard requirement for dried fish powder products (41–43) and corresponds to water activity values close to 0.65 which is in accordance with shelf stability requirements from a microbial point of view.

### 4.2 Mineral composition

The iron content of the fresh fish samples in this study is within the range of that reported in the FAO/INFOODS uFish 1.0 database, and similar to that reported by Nölle et al. (34) for fresh *Mpende* (0.41–0.44 mg/100 g) (Table 4). The dried, smoked, and powdered fish products have higher iron content per 100 g. The iron content of sun-dried, smoked and fish powder samples in this study was much lower than reported for smoked, dried, and powdered fish produced in Zambia (34, 36) and Malawi (37). The variations observed may be attributed to additional ingredients incorporated into the fish powders analyzed by Byrd et al. (36) or differing processing conditions. For instance, the fish powders analyzed for MAFOODS (37) were processed using laboratory-grade equipment, whereas the current study utilized local processing equipment. Overall, the data presented here indicate that small servings (10–25 g) of dried, smoked, and powdered fish products could contribute to dietary iron intakes in Zambia (nearly 5% for PLW and infants) which is of high bioavailability as an animal-source food (30). This could have important policy implications given the public health significance of iron deficiency in Zambia, with prevalence of anemia caused by iron deficiency estimated at 58% in children aged 6–59 months. The prevalence of anemia is notably higher among younger children (aged 6–23 months) compared to older children (aged 24–59 months). Specifically, 77% of children aged 9–11 months and 12–17 months experience anemia due to iron deficiency (2). There are well documented negative effects of iron deficiency on physical and cognitive development, pregnancy outcomes, morbidity, and mortality.

The results for zinc presented in this study (Table 4) fall within the range reported by FAO/INFOODS for fresh fish and seafood (77), and are comparable to those found in fresh *Mpende* and *Amatuku* analyzed by Nölle et al. (34). However, for smoked *Mpende* and fish powder samples in this study, zinc concentration is much lower than that reported for smoked *Mpende* (4.88 mg/100 g) and fish powder (0.96 mg/10 g), respectively (34, 36). The differences in zinc content between fish powders observed in this study and those reported by Byrd et al. (36) may also be attributed to the inclusion of additional ingredients in the fish powders. All dried, smoked, and powdered fish products analyzed in this study contribute up to 5% of RNI for infants and PLW (Figures 3, 4). Recent estimates indicate that zinc deficiency affects approximately 17% of the global population, with even higher prevalence rates observed in Africa at 24% (72, 73). Zinc present in animal-source foods, such as dried, smoked, and powdered fish products, exhibits high bioavailability and can significantly contribute to dietary zinc intake (30). Furthermore, incorporating animal-source foods such as fish into meals that primarily consist of plant-based foods can improve the absorption of nutrients like iron and zinc from these plant-based sources (74).

The results for calcium content of fresh fish products included in this study are within the range of fish and seafood reported by FAO/INFOODS. Our results are higher than those reported by Nölle et al. (34) for fresh *Mpende* (48.98–58.55 mg/100 g), smoked *Mpende* (323.06 mg/100 g), and fresh *Amatuku* (1,055.05 mg/100 g). Calcium content of the fish samples tested in this study was high possibly due to inclusion of the fish bones as edible parts. Calcium content of dried, smoked, and powdered fish products was outside of the ranges reported by FAO/INFOODS since they notably did not include dried and smoked fish products in their database. The calcium content was only slightly lower than the values reported for fish powders by Byrd et al. (36) and comparable to those reported for fish powder in the Malawi food composition table. All the dried and smoked fish products would contribute 10–15% of RNI for calcium for PLW and infants, while fresh fish samples contribute 13–22% (Figures 3, 4). However, the fresh fish samples would still need to be prepared for consumption, and depending on the cooking method and parts consumed, the nutrient content consumed will differ. A nutrient gap assessment conducted in Zambia identified possible deficiencies in calcium intake for children, particularly during the complementary feeding period. The report suggests that small, dried fish could help address these micronutrient gaps (44). Calcium deficiency increases the risk of rickets, but broader health implications of calcium deficiency in young children are poorly understood (45, 46). In many higher-income countries, dairy products tend to be the primary source of dietary calcium. However, this is not the case in Zambia where milk consumption is much lower than recommended levels (45). The most recent Zambia National Food Consumption and Micronutrient Status Survey (conducted in 2020) found that 97% of children aged 24–59 months were calcium deficient, as well as 100% of adolescent girls (10–14 years), and all groups of women (non-pregnant non-lactating, lactating, and pregnant women) (47). The data presented in this study further support the conclusion that in Zambia, local fish products consumed or processed in their whole form, with bones are a significant source of highly bioavailable dietary calcium (36, 44).

Selenium content of foods varies significantly according to surrounding environmental conditions (24) and species differences (48). The selenium content in fresh fish analyzed here ranged from 1.55–1.74 μg/100 g. These values are lower than data on selenium content of fresh fish as reported in FAO/INFOODS and Nölle et al. (34). Their data for fresh *Mpende*, smoked *Mpende*, and fresh *Amatuku* were 23.57–50.22 μg/100 g, 105.64 μg/100 g, and 24.40 μg/100 g, respectively. In general, higher levels of selenium are found in marine fish than in freshwater fish (49). All fish samples would provide less than 5% of RNI for selenium for PLW and infants (Figures 3, 4) based on the serving sizes used in this study. Other studies in the region which analyzed fish powders (36, 37) did not include selenium content of whole dried, smoked, or powdered fish, however studies from other regions have noted sufficient levels of selenium in dried whole fish to meet dietary needs (50, 51).

### 4.3 Vitamin composition

Based on RNIs for PLW and infants, the quantities of vitamin A identified in fish products analyzed in this study (Table 5) do not make a significant contribution to vitamin A intake. However, these are the first known results of vitamin A content of dried, smoked, and powdered fish products in the region, as identified studies from Zambia (34, 36) and the Malawi Food Composition table did not publish estimates of vitamin A content. Whole fish (particularly including the eyes) are typically rich in vitamin A, however a recent study from Cambodia found that vitamin A content of small freshwater fish is rapidly degraded in the drying process (52), due to sunlight (50). On the other hand, here we found that kiln-smoked fish retained more vitamin A, suggesting that faster drying in the kiln (in relation to sun-drying) has a positive effect on retention of vitamin A. This is supported by other studies, which have found that degradation of vitamin A in fish is more dependent on sunlight than on high temperatures (50).

There is limited data on vitamin B12 in fish and seafood available for comparison in the literature. In the FAO/INFOODs uFish 1.0 database, vitamin B12 content of fresh fish and seafood ranges from 0.56 to 54 μg/100 g which is consistent with results reported in Table 5 of this study. Nölle et al. (34) reported higher vitamin B12 content for fresh *Mpende* (0.99–1.40 μg/100 g) and smoked *Mpende* (4.28 μg/100 g) than our study found, while their analysis of *Amatuku* did not show results for vitamin B12. For PLW and infants, 7 of the fish products tested could contribute a small percentage (1–3%) of the daily RNI in a standard portion. This is the first analysis of vitamin B12 composition of dried, smoked, and powdered fish as similar studies from Zambia and Malawi did not include results of B12 analysis (36, 37). Vitamin B12 deficiency has been recorded in 87% and 95% of Zambian children under 5 years and women, respectively (53). As dietary sources of vitamin B12 only include animal-source foods, for which fish plays a significant role in Zambia, increased consumption of fish is a potential food-based strategy to prevent vitamin B12 deficiency. Given the low quantities of vitamins A and B12 found in fish and fish products in this study, fish should be consumed as a part of a healthy diet, including other animal source foods which have higher bioavailability of vitamin B12 (54–56) and vitamin A, or fruits and vegetables rich in beta-carotene.

Very limited data on vitamin D in fish and seafood are available for comparison in the literature. Vitamin D estimates for fresh, or dried, smoked, or powdered fish are not included in FAO/INFOODS uFish 1.0, the Malawi Food Composition Tables nor Byrd et al. (36). Vitamin D2 and D3 estimates are included in studies from other regions such as Bangladesh (24), Australia (57) and the United States of America (58), all of which show greater D2 and D3 content than the total vitamin D content reported in this study (Table 5). Given that the recommended nutrient intake (RNI) of total vitamin D is 5 μg/day for PLW and infants, all fish products tested in this study contribute minimally to dietary vitamin D intakes, accounting for 1–4% of the RNI for PLW and infants. Although vitamin D2 is understood to be found in plant-source foods, particularly yeasts and fungi, there is some evidence of vitamin D2 in microalgae and zooplankton, which may account for its presence in fish if these are part of their diet (59). This is the first known analysis of vitamin D content in the two species included in this study, in various forms (fresh, dried, smoked, and powdered). However, that further analysis of vitamin D2, D3 and total vitamin D, is recommended.

There is evidence of rapid onset of thermal degradation and loss of heat-sensitive vitamins such as vitamin E after 5 min of heat treatment (60). Although all processed fish samples analyzed in this study underwent some form of heat treatment, it is notable that the enhanced processing techniques (solar tent drying and smoking kiln) yielded higher vitamin E content compared to traditional methods. This suggests that minor advancements in fish processing can significantly enhance the retention of vitamin E in fish products. Very limited data on *α*-tocopherol and total vitamin E content in fish and seafood are available for comparison in the literature or in global databases. In the Malawian food composition database, total vitamin E content ranges from 0.50–3.98 mg/100 g for various smoked, grilled, dried and fresh fish products, which is broadly consistent with the total vitamin E content of fish products presented here. The Australian food composition database, α-tocopherol content of fish and seafood ranges from 0.1 to 4.2 μg/100 g which is broadly consistent with results presented for the one fish sample in which α-tocopherol was detected in this (“(57)). This is the first time the vitamin E content of fish species in Zambia has been presented.

Although our results for fatty acids ALA, LA, EPA, and DHA are lower than those reported by Nölle et al. (34) for *Tilapia rendalli*, they are within the range of data found in the FAO/INFOODs uFish 1.0 database for fish and shellfish for total PUFAs (0.05 g/100 g-0.55 g/100 g). Content of essential fatty acids in fish species differs by species, habitat and diet of the fish (61). While samples collected by Nölle et al. (34) were collected from multiple water bodies across Zambia, samples in our study were collected from one water body. All fish samples tested in this study contributed 6–33% of daily DHA requirements for WRA or infants (7–12 months). This is of particular interest given the growing body of literature on the role of fatty acids in growth and development during the first 1,000 days, and specifically, the role of DHA in normal retinal and brain development (62). The data presented here indicate that common fish species and products (dried and smoked fish) in Zambia, as well as innovative products like fish powders are a good dietary source of fatty acids and should be considered in food-based interventions to optimize growth and development during the first 1,000 days. It is also recommended that the fatty acid composition of other commonly consumed fish species be analyzed.

### 4.4 Significance of this study for policies in Zambia

The recently published Food Based Dietary Guidelines for Zambia promote the consumption of fish as a protein-rich food, and small fish such as “kapenta” (*Limnothrissa miodon*) as a calcium-rich food, as the fish is consumed without deboning (20). However, the Zambia Food Composition tables (19) lack nutrient content details on whole small fish. This study provides evidence of the nutrient composition of two fish species that are commonly consumed in Zambia, in their whole, fresh, dried, and powdered forms. Our analysis shows that these freshwater species are rich in calcium and selenium. Additionally, dried, smoked, and powdered fish products processed using simple technologies can make significant contributions to DHA requirements. This study also provides evidence that small improvements to technologies used for drying and smoking fish may have some effect on improving the nutrient content of fish products, particularly Vitamin E and Vitamin A through use of solar drying tents and improved fish smoking kilns. However, these technologies would need to be further tested to provide statistically significant data. The National Fisheries and Aquaculture policy implementation plan lists improved food and nutrition security as its first outcome, as well as improved value chains, highlighting the importance of the fisheries post-harvest sector and fish processing in ensuring that fish products reach consumers (18).

## 5 Conclusion

This study has provided evidence that several fish products processed from two common species in Northern Zambia (*Tilapia rendalli* and *Tilapia sparmanii*), can contribute to RNIs for nutrients of public health significance such as calcium, iron, selenium, and essential fatty acids. The results demonstrated that from a nutritional perspective, fish from inland fisheries (particularly small fish), and their products such as fish powder made using locally available technologies, represent an opportunity to provide micronutrients to vulnerable population groups such as PLW and infants within their first 1,000 days of life. This can at least be partially attributed to the way in which small fish are consumed (with head and bones, rich in micronutrients). Processing of these fish can extend their storage life and stabilize consumption throughout periods of lower fish availability, contributing to more sustainable fish supply chains. Improved processing methods can contribute to environmental sustainability by reducing fish loss and waste while also offering a socially and economically sustainable solution to combating malnutrition by offering a way to introduce fish to infants and young children in complementary feeding. Scaling introduction of cost-effective technologies such as solar drying tents and improved kilns may have some improvement on nutrient retention in dried and smoked fish. In addition, given difficulties experienced in this study with producing fish powder using traditional technologies like the mortar and pestle, scaling the introduction of hammer mills for fish powder production can reduce the burden on fish processors and reduce loss of nutritious parts such as bones that are not easily grinded during fish processing. In combination with these supply-side interventions, demand-side interventions can promote fish powder through national complementary feeding programs and consumer awareness campaigns on safe storage and preparation of fish for infants. This supports the compelling argument that to effectively target malnutrition and reach sustainable development goals such as zero hunger (SDG2) and sustainable production and consumption (SDG 12), resources should be directed towards investments in the fisheries post-harvest sector.

## Figures and Tables

**Figure A1:**
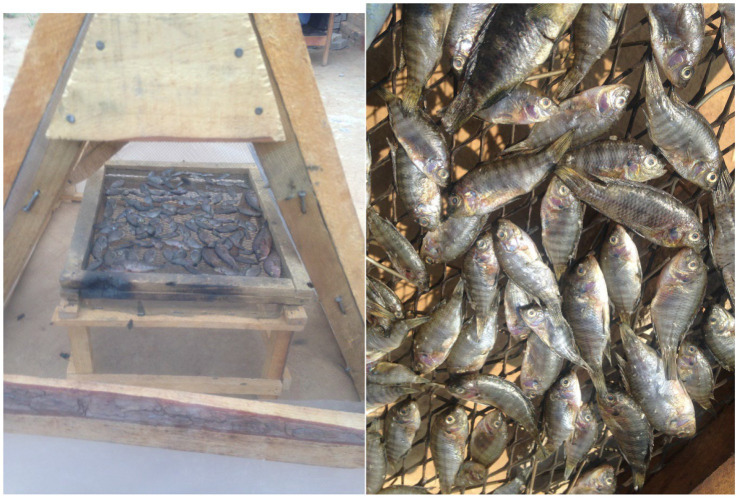
*Amatuku* (Tilapia sparmanii) drying in the solar tent (left) and open sun-drying (right).

**Figure A2:**
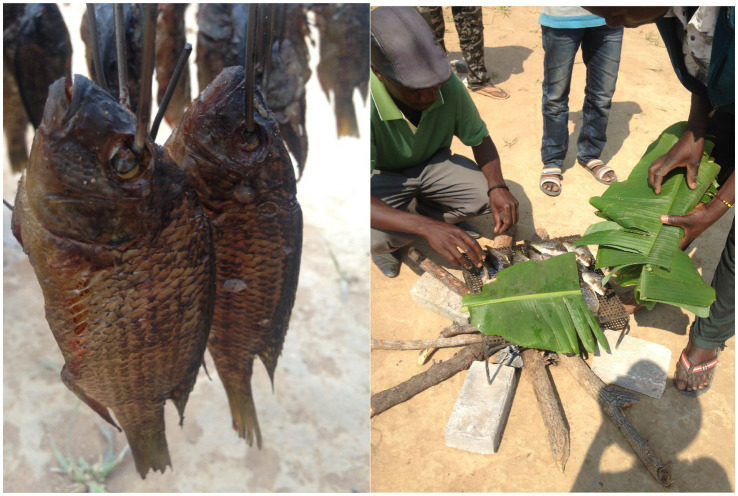
*Mpende* (*Tilapia rendalli*) hung from racks to be put in the smoking kiln (left) and smoked using the *ubusani* method—on a grill over open flame, covered with banana leaves (right).

## Data Availability

The original contributions presented in the study are included in the article/Supplementary material, further inquiries can be directed to the corresponding author.

## Footnotes

^1^Survey data is available through the FAO/WHO GIFT platform. Consumption data was used for freshwater fish and shellfish: fresh and processed (excluding dried). Conversion factors were used to convert weight of fresh fish serving size to dried fish serving size.

^2^Edible portions of each species were determined after the fish were cleaned (86% of the whole fresh fish weight for *Amatuku* and 77% for the whole fresh fish weight for *Mpende*), then further determined after drying or smoking the fish (resulting in the fish being reduced to 37–56% of its original weight as whole fresh fish). Much of this weight loss is attributed to reduction of moisture content, which results in a greater nutrient density per 100 g of dried / smoked / powdered fish products.
